# Striatal Dopamine Transporter Availability Is Not Associated with Food Craving in Lean and Obese Humans; a Molecular Imaging Study

**DOI:** 10.3390/brainsci11111428

**Published:** 2021-10-28

**Authors:** Jamie van Son, Katy A. van Galen, Anne Marijn Bruijn, Karin E. Koopman, Ruth I. Versteeg, Susanne E. la Fleur, Mireille J. Serlie, Jan Booij

**Affiliations:** 1 Department of Endocrinology and Metabolism, Amsterdam University Medical Centers, 1105 AZ Amsterdam, The Netherlands; j.vanson@amsterdamumc.nl (J.v.S.); k.a.vangalen@amsterdamumc.nl (K.A.v.G.); a.m.bruijn@amsterdamumc.nl (A.M.B.); k.koopman@amsterdamumc.nl (K.E.K.); r.i.versteeg@amsterdamumc.nl (R.I.V.); s.e.lafleur@amsterdamumc.nl (S.E.l.F.); m.j.serlie@amsterdamumc.nl (M.J.S.); 2 Department of Radiology and Nuclear Medicine, Amsterdam University Medical Centers, 1105 AZ Amsterdam, The Netherlands

**Keywords:** food craving, obesity, dopamine transporter, SPECT

## Abstract

Brain dopamine signaling is essential for the motivation to eat, and obesity is associated with altered dopaminergic signaling and increased food craving. We used molecular neuroimaging to explore whether striatal dopamine transporter (DAT) availability is associated with craving as measured with the General Food Craving Questionnaire-Trait (G-FCQ-T). We here show that humans with obesity (*n* = 34) experienced significantly more craving for food compared with lean subjects (*n* = 32), but food craving did not correlate significantly with striatal DAT availability as assessed with ^123^I-FP-CIT single-photon emission computed tomography. We conclude that food craving is increased in obesity, but the scores for food craving are not related to changes in striatal DAT availability.

## 1. Introduction

Craving is defined as “a strong feeling of wanting something”. In the light of the current obesity pandemic, it may not come as a surprise that most dictionaries explain the noun “craving” with the use of an example of food, i.e.,: “a craving for chocolate” [1]. An ongoing craving for food is often argued to cause obesity, and includes emotional, behavioral and physiological aspects. Even though hunger, induced by an energy deficit, and food craving often occur simultaneously, they are not the same. Food craving can occur without nutritional deprivation and has been linked to dysregulated eating, obesity, low dieting success and eating disorders [2,3,4]. More specifically, the occurrence of craving for food has been estimated to account for 7–11% of the variance in body mass index (BMI) [5,6]. Over the past few decades, research has focused on how obesity affects the brain, and vice versa, and has aimed to elucidate the mechanisms behind the increased craving for food that some individuals with obesity experience.

A role for the brain dopamine system in the motivation to seek food has been well established [7,8,9,10]. More specifically, studies in rodents have shown that dopamine signaling is essential for the motivation to eat [11,12] and for food reward [13,14]. Dopamine release in areas such as the striatum—a core part of the mesolimbic reward system [15]—acts on dopamine D_1_ and D_2_ receptors, influencing motivation [16,17,18]. Furthermore, dopamine release has been suggested to act as a central caloric sensor that is required to balance energy intake with energy requirements [19]. In lean humans, food-induced striatal dopamine release correlated with the experienced meal pleasantness [20] and a stronger post-ingestive dopamine release was associated with a lower post-prandial craving for food [21]. These findings imply a role for an adequate dopamine response in the suppression of food cravings when metabolic needs have been met. However, in obese subjects, a blunted dopamine response to food, along with a decreased striatal dopamine D_2/3_ receptor availability, has been observed [22,23,24]. Altogether, dysfunctional dopaminergic signaling may promote a stronger craving for food in obesity.

The dopamine transporter (DAT), expressed in the membrane of dopaminergic neurons, is a transmembrane protein which is an important regulator of extracellular dopamine [25,26]. DAT regulates the reuptake of dopamine from the synapse into the presynaptic neuron, thus terminating synaptic dopamine signaling. A reduction in DAT function results in the delayed termination of phasic dopamine release and enhanced tonic dopamine [27]. Midbrain tonic dopaminergic activity has been demonstrated to track reward values [28]. Moreover, studies using DAT knockdown mice have reported enhanced motivation for food rewards [8,29]. Additionally, we previously observed a faster response to a visual food stimulus in humans with decreased striatal DAT availability [30]. Therefore, changes in DAT availability or function may contribute to the regulation of craving for food.

Although previous research did not reveal a consistent difference in striatal DAT availability between humans with a healthy BMI and humans with obesity [24], a difference in striatal DAT availability may contribute to the increased craving for food that some, but not all, individuals with obesity may experience. To ultimately develop new therapies specifically targeted to reduce pathological food craving, it is highly relevant to elucidate the pathophysiology behind the obesity-associated increased craving for food.

In summary, in obesity, a stronger craving for food and disruptions in the dopaminergic system have been reported. However, currently, little is known on whether changes in the dopaminergic system in humans with obesity are associated with increased food craving and knowledge on the underlying mechanisms is scarce. Therefore, we here investigated whether craving for food is increased in obesity, and whether craving is associated with striatal DAT availability. We hypothesized that: (i) scores for food craving are higher in subjects with obesity compared to lean subjects; (ii) striatal DAT availability does not differ between subjects with obesity and lean subjects; and (iii) food craving scores are negatively associated with striatal DAT availability, particularly based on previous observations as described above [8,29,30].

## 2. Materials and Methods

### 2.1. Participants

Subjects participated in molecular neuroimaging studies conducted at the Amsterdam University Medical Centers (Amsterdam UMC, Amsterdam, The Netherlands) between February 2011 and October 2017 [31,32,33,34]. Subjects were recruited from the general population through local advertisements. Criteria for participation in these studies included age ≥ 18 years, a BMI < 25 kg/m^2^ or ≥30 kg/m^2^ and a stable weight for 3 months. Exclusion criteria included: the use of any medication (except for thyroid hormone, antihypertensive, and/or lipid-lowering drugs), history of a neuropsychiatric or eating disorder, shift work, irregular sleep pattern, alcohol and/or drug abuse, and smoking. Subjects underwent a medical evaluation, including medical history, physical examination, and blood tests, before study participation. Consecutive subjects were eligible for the present study if they underwent single-photon emission computed tomography (SPECT) imaging to measure striatal DAT availability after an overnight fast and completed the General Food Craving Questionnaire-Trait (G-FCQ-T). All procedures were approved by the Amsterdam UMC, location Academic Medical Center, medical ethics committee and all subjects provided written informed consent in accordance with the Declaration of Helsinki.

### 2.2. Anthropometric Measurements

Demographic and anthropometric parameters were measured and documented by trained medical doctors. Body composition was measured by bio-electrical impedance analysis (BF-906; Maltron, Rayleigh, UK). Subjects’ height and weight were used to calculate BMI. Following the definition from the World Health Organization, a lean individual was defined by a BMI of 18.5–24.9 kg/m^2^ and subjects with obesity by a BMI of ≥30 kg/m^2^ [35].

### 2.3. Brain Single-Photon Emission Computed Tomography (SPECT) Imaging

To measure DAT binding potential, subjects underwent SPECT imaging after an overnight fast. They were pre-treated with potassium iodide to limit thyroid uptake of free radioactive iodide. Imaging was performed 3 h after intravenous bolus injection of approximately 100 MBq ^123^I-FP-CIT [36] (specific activity, >750 MBq/nmol; radiochemical purity, >98%, produced according to good manufacturing practices criteria at GE Healthcare, Eindhoven, The Netherlands). SPECT imaging was performed on one of two brain-dedicated cameras: (i) the InSPira HD system (Neurologica, Boston, MA, USA), acquisition time per slice 180 s, slice thickness 4 mm; or (ii) the 12-detector, single-slice brain-dedicated scanner (Neurofocus 810; Strichman Medical Equipment, Cleveland, OH, USA), acquisition time per slice 210 s, slice thickness 5 mm. SPECT images were corrected for attenuation, and reconstructed as previously described [37,38]. We have previously demonstrated that striatal ratios are closely correlated between both cameras [37].

### 2.4. Region-of-Interest (ROI) Analysis

Striatal DAT availability was quantified automatically with the Brain Registration and Analysis Software Suite (BRASS; HERMES Medical, Sweden). BRASS registers the SPECT data to a template containing pre-specified regions-of-interest (ROI) and the occipital cortex. The occipital cortex was used to quantify nonspecific activity [39]. The specific-to-nonspecific binding potential (non-displaceable binding potential; BP_ND_, i.e., DAT availability) for the ROIs was consecutively calculated using the following formula: BP_ND_ = (mean ROI binding − mean occipital cortex binding)/mean occipital cortex binding. ROIs were the bilateral striatum, and its subregions: the caudate nucleus and putamen.

### 2.5. General Food Craving Questionnaire-Trait

The Dutch version of the G-FCQ-T is a validated self-report questionnaire to measure an individual’s stable experience of craving for food [40]. The questionnaire consists of 21 items, each relating to one of the four subscales: “preoccupation with food” (i.e., obsessively thinking of food), “loss of control” (i.e., difficulty in regulating eating behavior), “positive outcome expectancy” (i.e., the anticipation of positive reinforcement as the result from food intake) and “emotional craving” (i.e., emotions induce a stronger craving for food). Participants indicate to which degree a statement is applicable to them (in general, as a trait) on a 6-point Likert scale (1 = never, 6 = always). Mean scores were calculated for each subscale and the questionnaire in total. Higher scores reflect more frequent food craving experiences.

### 2.6. Statistical Analysis

Categorical variables are presented as count and percentage (%). Continuous variables are presented as mean ± standard deviation (SD) and median (interquartile range (IQR)) for parametric and nonparametric data, respectively. Unpaired *t*-tests, Mann–Whitney U tests and Fisher’s exact tests were used to compare groups as appropriate. One-way analyses of covariance (ANCOVA) were used to assess differences between groups while adjusting for the effect of a covariate. Partial regression analyses were used to assess correlations while adjusting for a covariate. Results with a *p*-value < 0.05 were considered statistically significant. Analyses were performed using IBM SPSS statistics version 26 (SPSS, Inc., Chicago, IL, USA).

## 3. Results

### 3.1. Study Subjects

**Figure 1:**
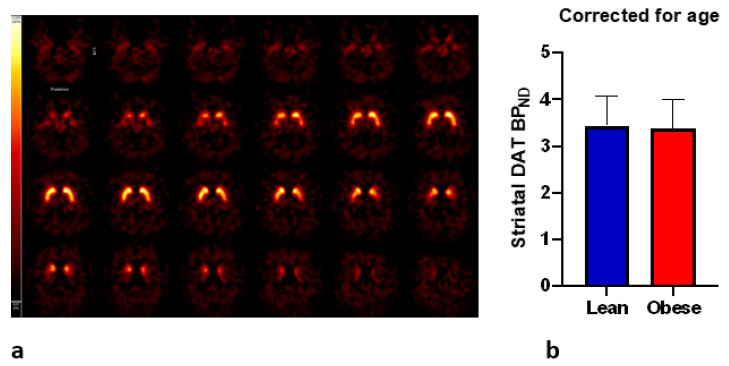
Striatal DAT BP_ND._ (**a**) A representative example of SPECT image of the brain (transversal slices) acquired 3 h after administration of ^123^I-FP-CIT in a lean subject. (**b**) Striatal DAT BP_ND_ (mean ± SD) did not differ significantly between lean subjects and subjects with obesity after controlling for age (one-way ANCOVA).

We included 66 subjects with a median age of 50 (23–63) years and 89.4 percent were male (Table 1). Thirty-two (48.5%) had a healthy bodyweight (BMI < 25 kg/m^2^) and the remaining 34 subjects had obesity (BMI ≥ 30 kg/m^2^). As striatal DAT availability declines with aging [41], and age differed between the lean subjects and the subjects with obesity, we adjusted for age in the comparison of striatal DAT availability between the two groups. Striatal DAT availability did not differ significantly between lean subjects and subjects with obesity after controlling for age (*p* = 0.681, Figure 1). Additional subregional analyses did also not reveal a statistically significant difference in DAT availability in the caudate nucleus or putamen between lean subjects and subjects with obesity (Supplemental Figure S1).

### 3.2. BMI and Craving

**Figure 2:**
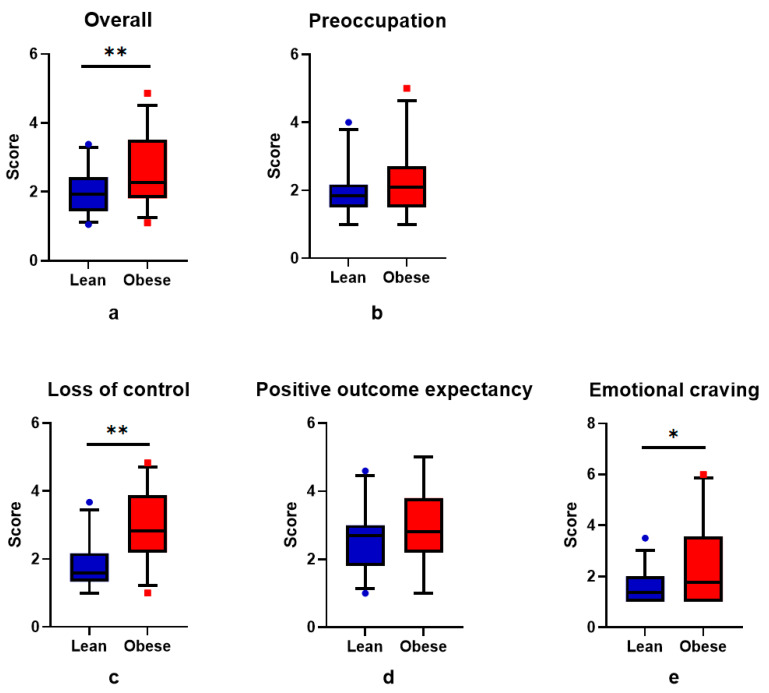
Boxplot of G-FCQ-T (**a**) overall score, and the subscale scores (**b**) “preoccupation”, (**c**) “loss of control”, (**d**) “positive outcome expectancy”, (**e**) “emotional craving” between lean subjects (blue) and subjects with obesity (red). Data are median 5th and 95th percentile. * *p* < 0.05 and ** *p* < 0.01 for Mann–Whitney *U* test.

First we explored the difference between lean subjects and subjects with obesity for scores on the G-FCQ-T questionnaire overall and its subscales (Figure 2). There was a statistically significant difference between lean subjects and subjects with obesity on the overall score (1.93 [1.45–2.38] vs. 2.27 [1.81–3.48]; *p* = 0.007) and the subscales “loss of control” and “emotional craving” (1.58 [1.33–2.17] vs. 2.83 [2.20–3.83]; *p* < 0.001 and 1.38 [1.00–2.00] vs. 1.78 [1.00–3.50]; *p* = 0.041, respectively). There was no significant difference between groups on the subscales “preoccupation” and “positive outcome expectancy” (1.83 [1.50–2.17] vs. 2.08 [1.50–2.70]; *p* = 0.231 and 2.58 (0.94) vs. 2.98 (1.10); *p* = 0.118, respectively). The higher score for the G-FCQ-T overall and the subscales “loss of control” and “emotional craving” indicates a stronger craving for food in subjects with obesity.

### 3.3. DAT and Craving

**Figure 3:**
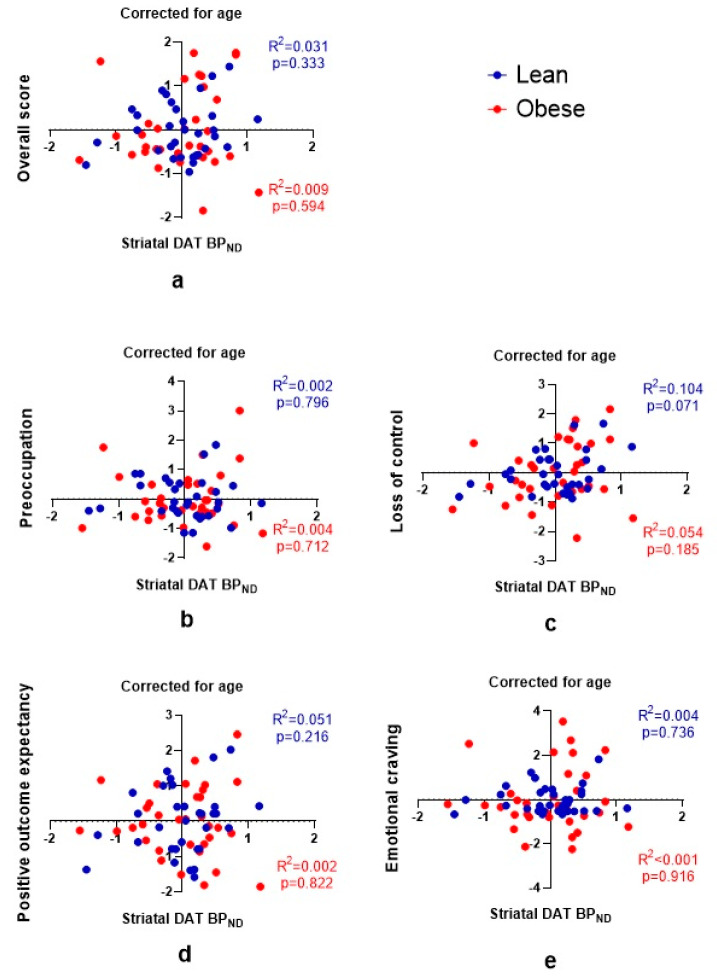
Partial regression plots showing no linear relationship between striatal DAT availability and G-FCQ-T scores for (**a**) the overall score, and the subscale scores (**b**) “preoccupation”, (**c**) “loss of control”, (**d**) “positive outcome expectancy”, (**e**) “emotional craving”, after adjusting for age in lean subjects and subjects with obesity. Blue dots: lean subjects; red dots: subjects with obesity.

Next we explored if craving for food is associated with striatal DAT availability in lean subjects and subjects with obesity. Using partial correlations to take into account the effect of the covariate “age”, we observed no linear correlation between striatal DAT availability and the G-FCQ-T overall score or its subscales in lean subjects or subjects with obesity (Figure 3). Additionally, analysis of the subregions the caudate nucleus and the putamen confirmed this lack of correlation (Supplemental Figure S2). These results show that a stronger craving for food was not associated with a lower or higher striatal DAT availability.

## 4. Discussion

Data from the present study confirm that subjects with obesity have an increased overall food craving compared to lean subjects, which is caused by an increased loss of control and emotional craving. Next, our data confirm the lack of difference in striatal DAT availability between humans with normal weight and obesity as previously reported [32,33,42,43,44]. We hypothesized that an increase in food craving would be related to a difference in DAT availability. However, we did not observe such an association.

One of the most often used instruments for the assessment of food craving is the self-reported measure G-FCQ-T, which refers to an individual’s stable experience of craving for food. The observed stronger craving for food in subjects with obesity is consistent with previous studies [45]. We here specifically report higher scores for the subscales “loss of control” and “emotional craving”, which may indicate that subjects with obesity experience a stronger tendency to crave food due to negative emotions and experience more difficulties in controlling eating behavior, respectively. Interestingly, we previously reported that the scores on these two subscales decrease following bariatric surgery [46], which suggests that these aspects of food craving are at least partially reversible. Finally, subjects with obesity did not score higher on the G-FCQ-T subscales “preoccupation” and “positive outcome expectancy”. Subjects with obesity may therefore not think about food and eating more obsessively and do not expect stronger positively reinforcing effects of food, respectively.

In line with previous studies [32,33,42,43,44], we did not find any difference in striatal DAT availability between lean subjects and subjects with obesity. However, changes in striatal dopamine D_2/3_ receptor availability are particularly pronounced in humans with a BMI > 40 kg/m^2^ [24]. As we included only 4 subjects with a BMI > 40 kg/m^2^, we cannot exclude BMI-associated changes in striatal DAT availability occurring with a further progression of obesity to a BMI > 40 kg/m^2^. Moreover, all included subjects were imaged after an overnight fast and we have recently shown that fasting-induced changes in striatal DAT availability may, in part, be explained by peripheral metabolic signals of fasting [32]. However, this dopaminergic response to fasting did not differ between lean subjects and subjects with obesity and has therefore unlikely confounded our findings [32]. In addition, we have earlier reported striatal DAT availability to change in a weight loss-independent, but meal timing-dependent manner in men with obesity following a diet-intervention [34]. Hence, striatal DAT availability may be influenced by obesity-related factors other than BMI per se, such as meal timing and other dietary choices [34,47,48].

As we previously observed a faster response to a visual food stimulus in humans with decreased striatal DAT availability [30], we hypothesized that decreased DAT availability would be associated with a stronger craving for food. A decreased DAT availability could result in higher tonic dopamine levels in the synapse, which has been associated with a stronger motivation for food [29]. Although we did observe a substantial variance in both the scores for food craving and striatal DAT availability, these parameters were not correlated in lean subjects or subjects with obesity. One explanation that may contribute to the absence of such a correlation could be that this questionnaire is a self-reported measure of food craving [49], which makes it possible for subjects to fill in socially desirable answers. In addition, the G-FCQ-T is a measure of craving in general and does not reflect the experienced food craving at the specific moment that SPECT imaging was performed [49]. Striatal DAT availability may therefore not be associated with food craving in general, but may be related to the intensity of momentary food craving. Another possibility is that mechanisms other than changes in striatal DAT availability may contribute to the increased craving for food in subjects with obesity. More severe obesity (BMI > 40 kg/m^2^) has consistently been associated with decreased dopamine D_2/3_ receptor availability [22,24,50,51]. In addition, dopamine release has been shown to be blunted to food and other stimuli in subjects with obesity [24,50]. Decreased postprandial dopamine release has been associated with a stronger craving for food after a meal [21]. Therefore, changes in the central dopaminergic circuitry, other than DAT availability, may contribute to stronger experiences of food craving in subjects with obesity and it would be of interest to study these aspects of the dopaminergic system in relation to food craving in the near future. However, we have previously reported no correlation between changes in striatal D_2/3_ receptor availability and G-FCQ-T scores in subjects undergoing bariatric surgery [46]. Finally, since other neurotransmitters than dopamine are also involved in craving for food, variation in these neurotransmitters may also account for the obesity-associated increase in craving for food [52,53].

This study comprises a large, but heterogeneous, population. This is a strength but also a limitation. Lean subjects had a significantly younger age compared to the subjects with obesity. Because age is negatively correlated with striatal DAT availability [41], we controlled for the effect of the age difference for analyses that included striatal DAT availability. As scores on the G-FCQ-T do not correlate with age [49], the stronger craving for food in subjects with obesity is not likely caused by differences in age between the groups. Furthermore, even though all subjects were classified as healthy before inclusion, there are metabolic consequences of obesity which are not accounted for, such as low-grade inflammation and insulin resistance [54], both of which have been suggested to impair dopaminergic signaling [55,56]. We therefore analyzed the correlation between striatal DAT availability and food craving in both groups separately. Moreover, 89.4% of the subjects of this study cohort were male. Several studies have reported a sex difference in the reward system in both humans and rodents [57,58,59,60,61]. Due to the small amount of females in this present cohort, we do not expect differences in sex to affect the outcome measures. However, it is important to note that the observations from this study may not translate to a predominantly female population. Finally, to be able to study a large population, we included subjects from multiple studies that were performed on two different scanners. However, as we have previously demonstrated that striatal ratios are closely correlated between both cameras [37], and lean subjects and subjects with obesity were equally distributed between both cameras, it is unlikely that a difference between both cameras has significantly affected our findings.

## 5. Conclusions

In conclusion, we show that human subjects with obesity experience stronger food craving as measured with the G-FCQ-T, but these scores for food craving are not related to changes in striatal DAT availability. These results indicate a mechanism other than changes in striatal DAT availability per se to underlie the increased G-FCQ-T in humans with obesity.

## Figures and Tables

**Table 1:** Demographic and anthropometric characteristics of the included subjects (*n* = 66).

	Lean Subjects *n* = 32	Subjects with Obesity *n* = 34	*p*-Value
Sex, male (%)	32 (100)	27 (79.4)	0.007
Age (years)	23 [21,58]	54 [48,67]	<0.001
BMI (kg/m^2^)	22.9 [21.4–23.9]	33.1 [32.3–36.2]	<0.001
Fat percentage (%)	16.5 ± 5.8	33.4 ± 8.0	<0.001

Data are count (%), mean ± SD, or median (IQR) and compared by Fisher’s exact test, *t*-test or Mann–Whitney *U* test.

## Data Availability

The data presented in this study are available on request from the corresponding author.

## Supplementary Materials

The following are available online at https://www.mdpi.com/article/10.3390/brainsci11111428/s1, Figure S1: DAT BPND, Figure S2: Sub regional partial regression analysis adjusted for age between DAT BPND and G-FCQ-T scores.
