# Supplementary material for: Striatal Dopamine Transporter Availability Is Not Associated with Food Craving in Lean and Obese Humans; a Molecular Imaging Study
Source: Brain Sci. 2021 Oct 28;11(11):1428. doi: 10.3390/brainsci11111428 (PMC8615750; doi:10.3390/brainsci11111428)
Supplement: Supplementary file 1 [file brainsci-11-01428-s001.zip › brainsci-1409153-supplementary.pdf]

<u>SUPPLEMENTAL FIGURE S1</u>

DAT BP_{ND} in the Caudate and Putamen.

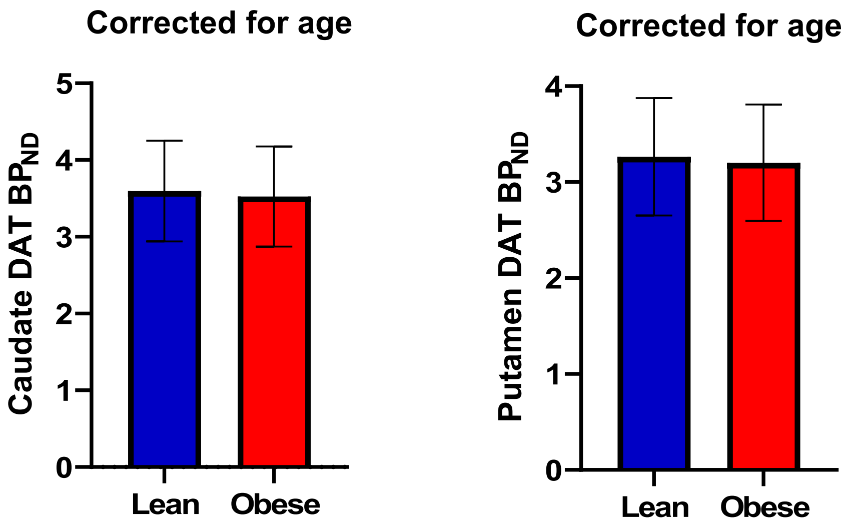

Supplemental figure S1. DAT BP_{ND} (mean ± SD) in the **(A)** Caudate and **(B)** Putamen did not differ significantly between lean subjects and subjects with obesity after controlling for age (one-way ANCOVA).

SUPPLEMENTAL FIGURES S2AB

Sub regional partial regression analysis adjusted for age between DAT BP_{ND} and G-FCQ-T scores.

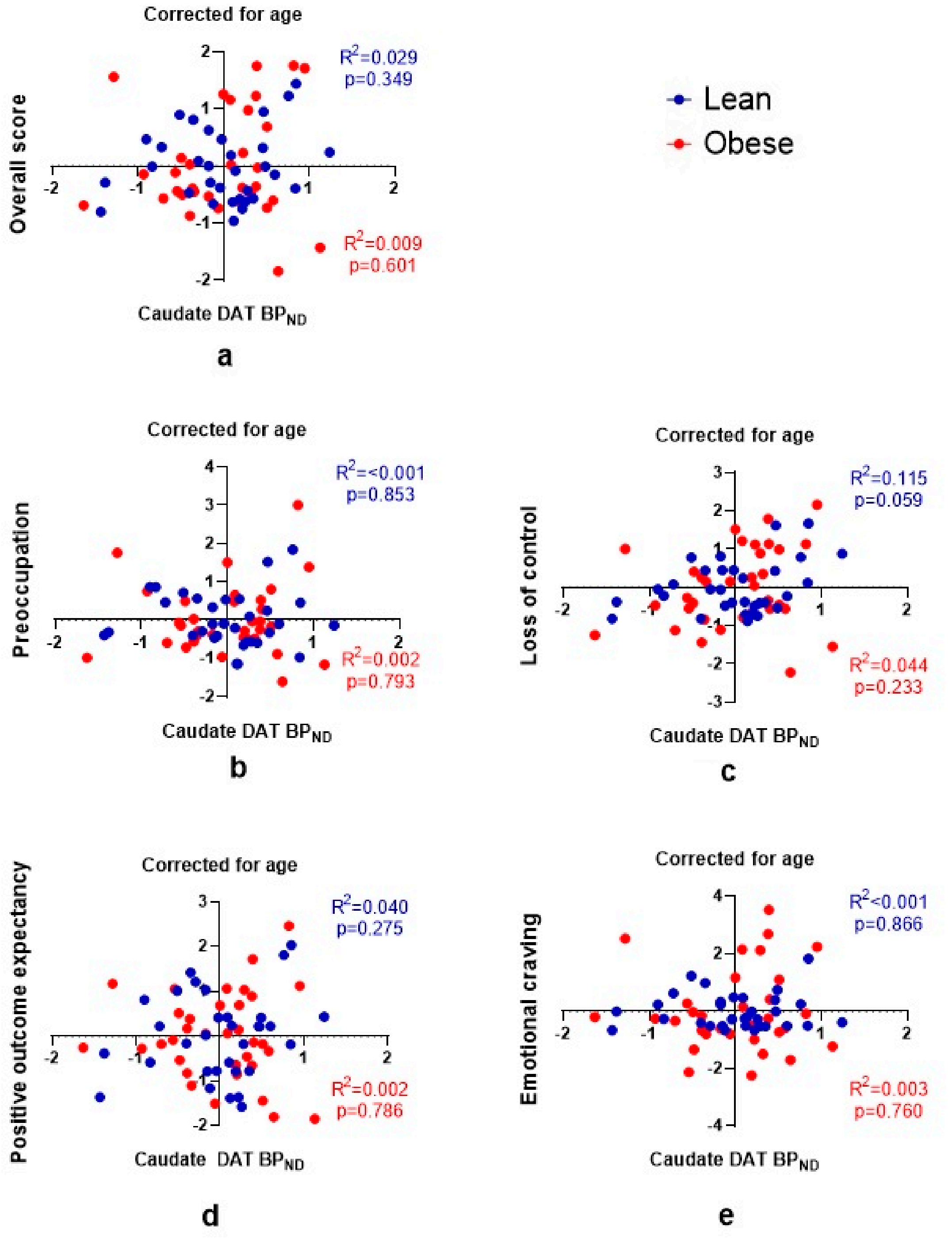

Supplemental figure S2 A. Partial regression plots showing no linear relationship between caudate DAT availability and G-FCQ-T scores for **(a)** the overall score, and the subscale scores **(b)** “preoccupation”, **(c)** “loss of control”, **(d)** “positive outcome expectancy”, **(e)** “emotional craving”, after adjusting for age in lean subjects and subjects with obesity. Blue dots: lean subjects; red dots: subjects with obesity.

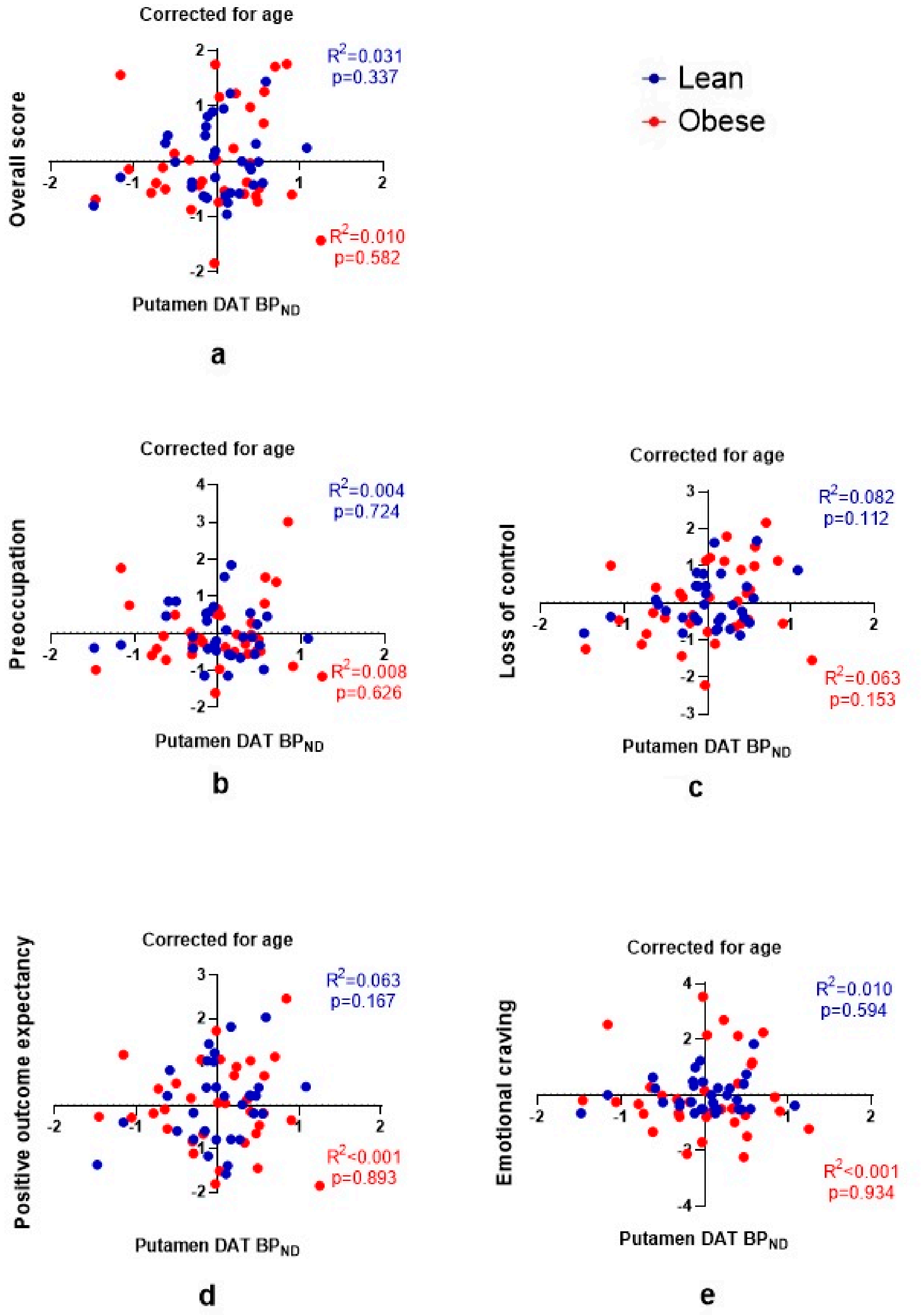

Supplemental figure S2 B. Partial regression plots showing no linear relationship between putamen DAT availability and G-FCQ-T scores for **(a)** the overall score, and the subscale scores **(b)** “preoccupation”, **(c)** “loss of control”, **(d)** “positive outcome expectancy”, **(e)** “emotional craving”, after adjusting for age in lean subjects and subjects with obesity. Blue dots: lean subjects; red dots: subjects with obesity.
